# Determination of clopidogrel effect in cats using point-of-care Plateletworks ADP and shipped samples for PFA-200 analysis in a clinical practice setting

**DOI:** 10.1177/1098612X241241404

**Published:** 2024-04-29

**Authors:** Matthew R Kornya, Anthony CG Abrams-Ogg, Shauna L Blois, R Darren Wood

**Affiliations:** 1 Department of Clinical Studies, Ontario Veterinary College, University of Guelph, Guelph, ON, Canada; 2 Department of Pathobiology, Ontario Veterinary College, University of Guelph, Guelph, ON, Canada

**Keywords:** Coagulation, clopidogrel, platelets, hematology

## Abstract

**Objectives:**

Clopidogrel is the recommended first-line antithrombotic in cats for a variety of conditions; however, it is ineffective in 15–20% of cats. The determination of clopidogrel effectiveness with platelet function assays has historically been limited to specialty centers; however, recent work has suggested that in-hospital or shipped analyses of samples may be feasible. The aim of the present study was to investigate the utility of an in-house analysis and shipping of blood samples collected in primary practices for the determination of clopidogrel effectiveness.

**Methods:**

Citrated blood samples were collected from cats receiving clopidogrel therapy by veterinarians in clinical practices across Canada, a median of 304.4 km from the reference laboratory (range 8–4425). Samples were analyzed in-house using Plateletworks ADP and shipped for remote analysis using PFA-200 P2Y and COL/ADP cartridges.

**Results:**

A total of 30 samples were collected from 25 cats. Of these, the percentage of samples analyzable for the presence or absence of the clopidogrel effect was 86% for Plateletworks ADP, 90% for PFA-200 P2Y and 87% for PFA-200 COL/ADP. There was no significant difference in the number of samples unable to be analyzed by each modality (*P* = 0.689) due to flow obstruction or other sample characteristics. The prevalence of absence of clopidogrel effectiveness on platelet function assays was 8% with the PFA-200 COL/ADP assay, 25% with the PFA-200 P2Y assay and 30% with the Plateletworks ADP assay.

**Conclusions and relevance:**

The results of this study confirm that samples of feline blood can be collected in clinical practices and shipped to a reference laboratory for PFA-200 analysis with a high rate of success, comparable to point-of-care analysis.

## Introduction

Clopidogrel is an antiplatelet agent used in human and veterinary medicine in the management of thromboembolism.^1,2^ However, based on the occurrence of thromboembolism during therapy and the results of platelet function tests, the drug may be ineffective in some patients.^2,3^ With respect to the latter, this is variably referred to as ‘clopidogrel resistance’ or ‘high-on-treatment platelet reactivity’, a situation in which platelets aggregate or react to agonists at a normal level, despite antiplatelet therapy.^3–6^ Clopidogrel resistance is difficult to precisely define as the drug dose needed to prevent thromboembolism for a specific condition in a specific species may not be known, and it is not known if the level of inhibition seen on platelet function assays correlates with clinical outcome.

Clopidogrel is a mainstay in the management of feline hypercoagulability, particularly in the prevention of aortic thromboembolism.^2,7^ Based on platelet function tests, ineffectiveness of the drug has been described in 15–20% of cats, though often with small sample sizes.^3–5,8–10^ Determining which patients are resistant has until recently been difficult outside of specialized practices due to the requirement for testing to be performed within hours of sample collection, preventing the analysis of samples shipped from other locations.^5,9^

Plateletworks (Helena Laboratories) detects platelet dysfunction by comparing platelet counts in EDTA with counts after exposure to an agonist. A platelet count in EDTA is measured on a hematology analyzer, and then measured again in a sample exposed to ADP, arachidonic acid or collagen, depending on the reason for testing. ADP is the agonist of choice for the detection of the clopidogrel effect.^11^ A standard hematology analyzer is used to determine platelet counts, and lower percent aggregations are associated with effective clopidogrel treatment.^9,11,12^ Two in-house analyzers have been validated for use with Plateletworks in cats for the detection of the clopidogrel effect.^9,13^ As such, Plateletworks remains the only commercially available system that may be used at point of care in general practice for the determination of the clopidogrel effect without the purchase of dedicated platelet function analyzers. Its lower cost and ease of use also make it an attractive choice. Previous attempts by our laboratory to validate sample storage and shipping for a Plateletworks analysis remotely for cats have not been successful.

The Platelet Function Analyzer 200 (PFA-200; Siemens Healthineers) evaluates platelet function in a physiologic fashion by drawing citrated whole blood under a vacuum through a channel and against a perforated membrane coated with various platelet agonists in a temperature-controlled environment.^14,15^ The analyzer returns a ‘Closure Time’ (CT) result as the time to stoppage of blood flow. Several test cartridges are available, including the original Collagen/ADP (COL/ADP) and its successor, the P2Y cartridge, which has been optimized for the detection of the clopidogrel effect. It has been demonstrated to reliably detect the clopidogrel effect in cats.^3,5,16^

Our laboratory has previously demonstrated that blood samples from healthy and clopidogrel-treated cats may be stored and shipped for a delayed PFA-200 analysis in an experimental model.^5^ In addition, we have validated the in-house hematology analyzer ProCyte Dx (IDEXX) with the Plateletworks system.^9^ For both of these studies, samples were collected in a tertiary care center and subjected to ‘mock’ shipping, without the inherent variability and challenges seen in primary care practice using a commercial courier service.

The purpose of this prospective study was to demonstrate the feasibility of in-house platelet function testing using Plateletworks ADP, and remote platelet function testing using the PFA-200, for the determination of the clopidogrel effect in clinical practices in a real-world setting.

## Materials and methods

Primary care and referral veterinary practices across Canada were recruited to the study using direct emails, social media posts and postings on the University of Guelph website. Ethical approval for this study was obtained from the University of Guelph Animal Care Committee (Animal Use Protocol 4732).

Cats were included if they had been receiving clopidogrel therapy for primary (therapy before a thrombovascular event) or secondary (therapy after a thrombovascular event has occurred) prevention of thromboembolic disease for a minimum of 4 days and were amenable to jugular venipuncture. Clinics were also required to have a ProCyte Dx Hematology analyzer (IDEXX) on-site, as well as the ability to facilitate sample shipping on the day of collection. Samples were required to be collected and shipped to arrive on Monday to Saturday. The exclusion criteria were the use of other anti-platelet drugs in a 1-month period before enrollment, anemia (hematocrit <25%) or thrombocytopenia (platelet count <100 ×10^9^/l). The concurrent use of anti-Xa drugs was permitted.

Clinics were offered reimbursement for the cost of a re-check examination, phlebotomy, gabapentin, sample shipping and complete blood count analysis. Upon confirming their interest, clinics were mailed a package containing an insulated shipping container, ice pack, sample tubes (EDTA-K2, 3.2% sodium citrate and Plateletworks ADP) and consent forms.

Owners were directed to administer gabapentin 100 mg PO the night before their appointment and 1–2 h before their appointment. Butorphanol 0.1–0.3 mg/kg IV was permitted to facilitate phlebotomy. On presentation to the veterinarian, it was recommended that phlebotomy be performed before other procedures to minimize patient stress during phlebotomy. We recommended that skin over the jugular vein be clipped, and 4% lidocaine gel applied for a minimum of 30 mins to further facilitate phlebotomy. Cats were manually restrained, and jugular venipuncture was performed with a 22 G, 1 inch needle attached to a 6 ml syringe with a target blood draw of 5.5–6 ml. If there were more than three redirections, stoppage of flow or redirection after initiation of flow, phlebotomy was stopped. If an insufficient sample was attained, venipuncture was repeated with the opposite jugular vein.

After phlebotomy, the needle was removed from the syringe and blood dispensed to the fill lines of the following opened tubes: first, one 1.1 ml Plateletworks ADP (20 µM ADP with 3.2% sodium citrate) tube, then one 0.5 ml EDTA-K2 (Greiner Bio-One) tube, and finally two 1.8 ml 3.2% sodium citrate Vacutainer tubes (Becton Dickinson Canada). Stoppers and caps were replaced, and tubes were then mixed with 15 full inversions.

The ADP aliquot was analyzed on the clinic’s on-site ProCyte Dx with a targeted time to analysis of 10 mins after collection (though allowing analysis in the first 30 mins based on previous findings)^9^ followed by an analysis of the EDTA sample approximately 2 mins later. Percentage aggregation (%Agg) was calculated according to manufacturer’s recommendations using the equation:

$$\begin{array}{l} \%Agg\;=\;\left(Platelet\;Count_{EDTA}-\;Platelet\;Count_{ADP}\right)\;/\;\\ \;\;Platelet\;Count_{EDTA}\times \;100\% \end{array}$$

The optimal cut-point for the determination of the clopidogrel effect using Plateletworks ADP on the IDEXX ProCyte Dx (21%) was previously determined using a receiver operating characteristic (ROC) analysis^9^ and was used for the determination of the clopidogrel effect in this population.

Citrated samples were packaged at the clinic in bubble wrap and packed into a Styrofoam container along with an ice pack, completed consent forms and Plateletworks results. Samples were shipped to the Ontario Veterinary College (OVC) by next-day air using a courier service of the clinics’ choosing.

On arrival at the OVC, samples were unpackaged within 4 h and allowed to acclimate to room temperature as confirmed by an infrared thermometer (Thermo Fisher Scientific). PFA-200 cartridges were warmed to room temperature for 20 mins. Samples were mixed 10 times by inversion and analyzed as per manufacturer recommendations,^17^ with both COL/ADP and P2Y cartridges using channel ‘A’ of the PFA-200 analyzer. P2Y cartridges were analyzed first, followed by COL/ADP cartridges. In cases of flow obstruction, enough samples were present to allow one additional analysis. If flow obstructions occurred with both P2Y and COL/ADP cartridges, preference was given to analyzing a duplicate P2Y cartridge. If duplicate flow obstructions occurred, closure curve and primary hemostasis component analyses were performed as previously described to determine the clopidogrel effect.^4^

The number of samples unable to be analyzed by each method was determined. Differences between the numbers of samples unable to be analyzed were investigated using χ^2^ analysis.

The optimal cut-point for determination of the clopidogrel effect using ‘mock’ shipped samples on the PFA-200 analyzer (176 s for P2Y, 120 s for COL/ADP) was previously determined using an ROC analysis^5^ and was used for determination of the clopidogrel effect in this population. Descriptive statistics for the prevalence of resistance to clopidogrel using the three assays were determined. Agreement between pairs of assays was investigated with Cohen’s kappa, and between all three assays with Fleiss’ kappa.

## Results

**Figure 1:**
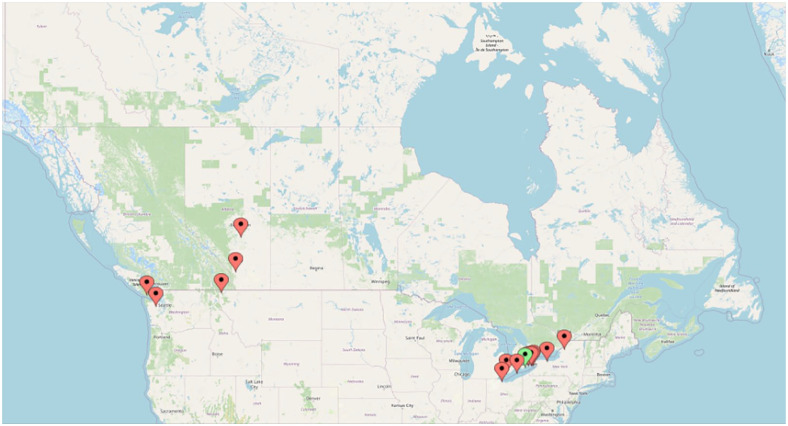
Locations of veterinary hospitals submitting samples of feline blood for PFA-200 analysis. Red markers = submitting hospitals; green marker = destination laboratory (source: Google Maps)

A total of 30 samples were collected from 25 cats at 18 unique practices across Canada. There were three clinics in British Columbia, two in Alberta and 13 in Ontario. The median distance from a submitting practice to the OVC was 304.4 km (range 8–4425). The largest number of samples (n = 7) came from a clinic 3338 km away. See Figure 1 for a map of locations. The median time from sample collection to analysis was 23 h (range 8–46).

There were 15 castrated male and 10 spayed female cats recruited. The median age was 4.6 years (range 1–13). The most common breed was domestic shorthair (16 cats), followed by Maine Coon (four cats), Sphynx (two cats), Devon Rex (one cat), British Shorthair (one cat) and Persian (one cat).

The most common reason for clopidogrel therapy was hypertrophic cardiomyopathy (HCM; 23 cases); other reasons included restrictive cardiomyopathy (RCM; two cases), protein-losing nephropathy (one case), immune-mediated hemolytic anemia (IMHA; two cases) and thyrotoxicosis (one case). All diagnoses were reported by the attending primary care clinician; in 12 cases, they were made by a cardiologist or internist. Six of the patients (five with HCM and one with thyrotoxicosis) had a history of a previous thrombus; the others had no history of thromboembolism. All cats were receiving 18.75 mg clopidogrel (1.83–6.0 mg/kg) at the initial sample collection. Two cats were re-tested after dose increase, and at this time point, they were receiving 35 mg (2.18–3.43 mg/kg).

Three cats received concurrent rivaroxaban 2.5–5 mg (0.59–1.04 mg/kg). One cat received concurrent apixaban 1.25 mg (0.21 mg/kg).

Plateletworks testing was intended to be performed on 29 samples. In one case, Plateletworks had already been performed and a sample was re-collected at a second visit for a PFA-200 analysis. Plateletworks results were available in 25/29 (86.2%) samples. Reasons for the lack of results included instrument malfunction (two samples), non-compliance with protocol (one sample) and lack of adequate sample volume collected (one sample). The median time from sample collection to initial Plateletworks analysis was 16.5 mins (range 8–26).

The PFA-200 analysis was intended to be performed on 30 samples. The sample was adequate for a PFA-200 analysis in 27/30 (90%) samples. Reasons for the lack of adequate sample included markedly delayed shipping (one sample), sample clotting (one sample) and sample freezing (one sample). Among the 27 samples analyzed on the PFA-200, duplicate flow obstructions using the COL/ADP cartridge occurred for one sample, so no closure time was reported (3.7% of 27 analyzed samples). This resulted in a total of 4/30 (13%) samples submitted for COL/ADP testing having no reported closure time. There were duplicate flow obstructions for three samples using the P2Y cartridge, so no closure time was reported (11% of 27 analyzed samples). None of these were from the same sample that had flow obstruction using the COL/ADP cartridge. This resulted in a total of 6/30 (20%) samples submitted for P2Y testing having no reported closure time. Of the samples that had flow obstruction, all P2Y samples were able to have the clopidogrel effect determined using the primary hemostasis component analysis; this was not possible in the COL/ADP sample.

Between the three assays, there was no sample that was unable to be analyzed at all. A breakdown of samples able to be tested is presented in Table 1. There was no significant difference in the number of samples unable to be analyzed by each modality (*P* = 0.689). The prevalence of resistance to clopidogrel was two (8%) cats on the PFA-200 COL/ADP assay, six (25%) cats on the PFA-200 P2Y assay and eight (30%) cats on the Plateletworks ADP assay. The agreement between all three assays was 0.67 (substantial agreement). The agreement between PFA-200 P2Y and COL/ADP was 0.18 (slight agreement), between PFA P2Y and Plateletworks (PW) it was 0.64 (substantial agreement) and between PFA COL/ADP and PW it was 0.19 (slight agreement).^18^

**Table 1:** Number of samples viable for analysis at each step of processing in cats enrolled from primary care veterinarians for determination of the clopidogrel effect

	Samples enrolled	Adequate blood collected	Samples analyzed	Non-flow obstructed	Presence or absence of clopidogrel effect determined
Plateletworks	29 (100)	27 (93)	25 (86)	N/A	25 (86)
PFA-200 P2Y	30 (100)	30 (100)	27 (90)	24 (80)	27 (90)
PFA-200 COL/ADP	30 (100)	30 (100)	27 (90)	26 (87)	26 (87)

Data are n (%)

N/A = not applicable

## Discussion

This study demonstrated that the determination of the clopidogrel effect in private practice is feasible, both by in-house and next-day remote analyses. The prevalence of resistance to clopidogrel in cats varies between studies but has generally been in the range of 10–30%.^5,8,19,20^ This is consistent with the 8–30% reported in the present study.

An in-house analysis with Plateletworks allows for a rapid turnaround time for results and removes the costs and risks associated with shipping (eg, late delivery, sample freezing). While the in-house analysis had the lowest reported number of results available, the causes of this were all likely avoidable (ie, the analyzer not working on the appointment day, not collecting adequate samples). The most significant limitation of this mode of testing is the requirement for a clinic to stock specialized sample tubes (Plateletworks ADP). While this may be feasible for a larger specialty center, cardiology service or corporate group, the minimum purchasable quantity of 25 and expiration date (<2 years) would make this uneconomical for many veterinarians.

The shipping of samples for remote analysis has the associated cost and labor of sample packaging and shipment, as well as the attendant risks due to poor sample handling. Currently, access to PFA-200 analyzers is limited; however, adoption of this technology by local reference laboratories would make the assays more available. We have demonstrated that the shipping of samples for a PFA-200 analysis is consistently feasible across several thousand kilometers, as such adoption by only a small number of reference laboratories would make nationwide access feasible.

Significant human data have suggested that a single test of platelet function is not sufficient to fully characterize platelet function or dysfunction in many situations.^21,22^ Veterinary data have also suggested that different tests of platelet function may provide variable results between individuals, in certain diseases and in the detection of anti-platelet effect.^13,23^ As such, the ideal testing protocol may involve a series of assays, and performing tests both in-house and at a remote laboratory may provide the most clinically relevant information.

Our previous work has validated the storage and shipping of samples for a PFA-200 analysis up to 27 h after collection.^5^ In this study, the median time to analysis was 23 h; however, samples were analyzed up to 46 h after collection. It is possible that some of the samples with longer delayed times to analysis may have had erroneous results; however, unpublished data from our laboratory have suggested that the holding of samples for up to 72 h leads to consistent closure times, although a higher rate of flow obstruction. Some of the patients in this study also received gabapentin as part of standard protocols in many clinics for stress reduction. There are some data in the human literature to suggest gabapentin may affect platelet function,^24,25^ which may have affected our results. This has not been noted in feline medicine, and we have demonstrated normal platelet function in many cats in our laboratory receiving gabapentin.^3,5,9^ Nonetheless, this is a potential limitation. In addition, this study did not evaluate the actual potential clinical outcome of clopidogrel resistance, that is, thrombosis. This would require large-scale longitudinal studies, which the results of the current study could help facilitate.

Of all samples collected, 86–90% were able to have the clopidogrel effect determined with any given assay, and all samples were able to have the clopidogrel effect determined using at least one of the three assays. Previous studies in our laboratory using point-of-care analysis within 4 h of sample collection had shown the rate of flow obstruction with the PFA-200 to be in the range of 10–20%,^4,5^ comparable with the results seen in this study.

## Conclusions

The results of this study confirm that samples of feline blood can be collected in clinical practices and shipped to a reference laboratory for a PFA-200 analysis with a high rate of success, comparable with results using a point-of-care analysis with Plateletworks ADP.
